# Electroacupuncture Attenuates Cerebral Ischemia and Reperfusion Injury in Middle Cerebral Artery Occlusion of Rat via Modulation of Apoptosis, Inflammation, Oxidative Stress, and Excitotoxicity

**DOI:** 10.1155/2016/9438650

**Published:** 2016-03-31

**Authors:** Mei-hong Shen, Chun-bing Zhang, Jia-hui Zhang, Peng-fei Li

**Affiliations:** ^1^The Second Clinical College, Nanjing University of Chinese Medicine, Nanjing, Jiangsu 210046, China; ^2^College of Basic Medicine, Nanjing University of Chinese Medicine, Nanjing, Jiangsu 210046, China; ^3^Department of Clinical Laboratory, Jiangsu Province Hospital of Traditional Chinese Medicine, Affiliated Hospital of Nanjing University of Chinese Medicine, Nanjing, Jiangsu 210029, China

## Abstract

Electroacupuncture (EA) has several properties such as antioxidant, antiapoptosis, and anti-inflammatory properties. The current study was to investigate the effects of EA on the prevention and treatment of cerebral ischemia-reperfusion (I/R) injury and to elucidate possible molecular mechanisms. Sprague-Dawley rats were subjected to middle cerebral artery occlusion (MCAO) for 2 h followed by reperfusion for 24 h. EA stimulation was applied to both* Baihui* and* Dazhui* acupoints for 30 min in each rat per day for 5 successive days before MCAO (pretreatment) or when the reperfusion was initiated (treatment). Neurologic deficit scores, infarction volumes, brain water content, and neuronal apoptosis were evaluated. The expressions of related inflammatory cytokines, apoptotic molecules, antioxidant systems, and excitotoxic receptors in the brain were also investigated. Results showed that both EA pretreatment and treatment significantly reduced infarct volumes, decreased brain water content, and alleviated neuronal injury in MCAO rats. Notably, EA exerts neuroprotection against I/R injury through improving neurological function, attenuating the inflammation cytokines, upregulating antioxidant systems, and reducing the excitotoxicity. This study provides a better understanding of the molecular mechanism underlying the traditional use of EA.

## 1. Introduction 

Stroke, a serious threat to human's health, is a major cause of death and disability in the world. Moreover, stroke has been a leading cause of death in China [1]. It is well known that stroke can be classified into ischemic stroke (IS) and hemorrhagic stroke (HS). Approximately, 87% of the stroke cases are ischemic in origin [2]. Although the exact molecular mechanisms of cerebral ischemia and reperfusion (I/R) injury are not fully known, several evidences indicate that excitotoxicity, oxidative stress, apoptosis, and inflammatory events that occur during cerebral ischemia are critical for the pathogenesis of tissue injury in IS [3–6]. So far, the thrombolytic agent tissue plasminogen activator (tPA) is the only FDA approved therapy for acute IS [7]. However, tPA has potential shortcomings including the risk of hemorrhagic transformation and narrow time window [8]. Therefore, new strategies that protect against ischemia are urgently needed.

Electroacupuncture (EA) was derived from traditional Chinese medicine and has been widely applied for treatment of many diseases as an alternative therapy method [9–11]. Increasing experimental evidences demonstrate that EA possesses many beneficial properties, such as neuroprotective, anti-inflammatory, and antiapoptotic effects in various animal models [12, 13]. EA has recently been shown to effectively exert neuroprotective effects on stroke patients [14] and in animal middle cerebral artery occlusion (MCAO) models [15]. Moreover, this neuroprotection is closely related to anti-inflammatory and antiapoptotic pathways [16–18]. Our previous studies demonstrated that EA at* Baihui* (GV20) and* Dazhui* (GV14) in MCAO rats protected cerebral cortical cells from injury by clearing away excessive oxygen free radicals [19]. However, the precise mechanism of EA's neuroprotective efficacy is still not well defined.

Therefore, the present study was conducted to investigate whether EA pretreatment or treatment at the* Baihui* (GV20) and* Dazhui* (GV14) acupoints improves cerebral I/R injury in rat. Furthermore, we elucidated the underlying mechanism which was related to the functional recovery.

## 2. Materials and Methods 

### 2.1. Animals and Groups

Eight-month-old specific-pathogen-free adult male Sprague-Dawley rats, weighing 280 to 320 g, were provided by SLRC Laboratory Animals (Shanghai, China) (certification no. SCXK (Hu) 2007-0005) and housed under diurnal lighting conditions (12 h light/dark cycle). The study conformed to the Guide for the Care and Use of Laboratory Animals published by the US National Institutes of Health (NIH publication no. 85-23, revised 1996), and the experimental procedures were consistent with the ethical requirements established by the Ethics Committee for Animal Experimentation of Nanjing University of Chinese Medicine. Rats were divided randomly into four groups: Sham-operation group (Sham), middle cerebral artery occlusion model-no treatment group (MCAO), EA pretreatment-MCAO group (EA pretreatment), and MCAO-EA treatment group (EA treatment).

### 2.2. Middle Cerebral Artery Occlusion Model

Rats were allowed free access to food and water but were fasted 12 h before surgery. The MCAO model was performed as described previously, with minor modifications [20]. During the procedure, room temperature was maintained at 27°C. Briefly, rats were anesthetized by intraperitoneal injection of 10% chloral hydrate (Abbott, Illinois, USA); the right common carotid artery, internal carotid artery, and external carotid artery were exposed through a ventral midline neck incision. The internal carotid artery was then isolated and coagulated, and the proximal common carotid artery was ligated. A 4-0 monofilament nylon suture (Beijing Sunbio Biotech Co. Ltd., Beijing, China) with a rounded tip was inserted into the internal carotid artery from the common carotid artery through the external carotid artery stump and gently advanced 18 to 20 mm to occlude the middle cerebral artery. Body temperature was maintained between 37 and 37.5°C by means of heating pad. After 2 h of MCAO, the suture was removed to restore blood flow (reperfusion). Sham-operation rats underwent identical surgery except that the suture was not inserted. During the experiments, Laser-Doppler Flowmetry (LDF) (MoorDRT4; Biopac Systems, Inc., Goleta, CA, USA) was used to monitor cerebral blood flow (CBF) before and after MCAO. Rats were anesthetized with 2% diethyl ether. The flexible 0.5 mm fiber optic probe was perpendicularly placed at 1 mm above the skull surface of the MCA territory (4 mm lateral and 2 mm posterior from bregma). This blood flow rate was maintained for at least 1 h, with the exception of the 0 h time-point. The MCAO model was considered successful only when the drop in cerebral blood flow was ≥70% of baseline during occlusion.

### 2.3. EA Stimulation

EA was applied to the acupuncture points* Baihui* (GV20) and* Dazhui* (GV14) with a pair of bipolar stimulation electrodes after placing the rats under intraperitoneal injection of 10% chloral hydrate (Abbott, Illinois, USA). Stainless acupuncture needles of 0.3 mm in diameter (HuaTuo, Suzhou Medical Appliance Factory) were applied to both* Baihui* (GV20) and* Dazhui* (GV14) acupoints in each rat (10 mm EA penetration depth, sparse-dense wave with a frequency of 2/15 Hz and a current intensity of 1~3 mA) using an electrical needle stimulator (WQ1002K, Electro-Acupuncture Equipment Company, China). For the EA pretreatment group, the animals underwent acupuncture once per day for a duration of 30 min for 5 consecutive days and then received MCAO. For the EA treatment group, after 2 h of MCAO, rats received EA stimulation for 30 min. Moreover, the stimulation parameters were the same as EA pretreatment.

### 2.4. Neurological Function Assessment

At 24 h after reperfusion, a neurological assessment of the rats in different groups was performed by a blind investigator using the 18-point scoring system reported by Garcia et al. [21]. The system consisted of the following six tests: (1) spontaneous activity, (2) symmetry in the movement of four limbs, (3) forepaw outstretching, (4) climbing, (5) body proprioception, and (6) response to vibrissae touch. The score given to each rat at the completion of the evaluation was the summation of all six individual test scores. Minimum neurologic score was 3 and maximum score was 18.

### 2.5. Quantification of Brain Water Content

The brain edema was determined by evaluating the brain water content according to the wet-dry method [22]. In brief, rats were decapitated under deep anesthesia with 10% chloral hydrate at 24 h of reperfusion and their brains were immediately acquired. A neutral filter paper was used to absorb and remove blood stains from the brain. The ipsilateral and contralateral hemispheres were dissected and the wet weight of the tissue was determined by an electronic scale (wet weight). Subsequently, the tissues were dried overnight at 105°C in a desiccating oven and the dry weight was obtained. Then, the brain water content was calculated using the following formula: brain water content (*g*) = (wet weight − dry weight).

### 2.6. Measurement of Infarct Volume

After neurological evaluation, rats were decapitated and the brains were rapidly removed and mildly frozen to keep the morphology intact during slicing. Infarct volume was measured as described previously [23]. In brief, the brain was rapidly dissected and sectioned into five coronal blocks in brain matrix with an approximate thickness of 2 mm and stained with 2% (w/v) 2,3,5-triphenyltetrazolium chloride (TTC) (Sigma, USA) for 30 min at 37°C followed by overnight immersion in 4% (w/v) paraformaldehyde. The infarct tissue area remained unstained (white), whereas normal tissue was stained red. The infarct areas on each slice were demarcated and analyzed by Image J software (National Institutes of Health, Bethesda, MD, USA). The infarct volumes were calculated via the method according to the following formula: ((total contralateral hemispheric volume) – (total ipsilateral hemispheric stained volume))/(total contralateral hemispheric volume) × 100%.

### 2.7. Histopathological Examination

Hematoxylin and eosin (HE) staining was performed to show the morphological features of injured neurons in the cerebral cortex. At 24 h after MCAO, rats were sacrificed and brains were fixed by transcardial perfusion with saline, followed by perfusion and immersion in 4% paraformaldehyde. Brains were then dehydrated in a graded series of alcohols and embedded in paraffin. A series of 5 *μ*m thick sections were cut from the block. Finally, the sections were stained with HE reagents for pathological histological examination. The slices were observed and photographed with an Olympus BX50 microscope (Tokyo, Japan).

### 2.8. Transmission Electron Microscopy (TEM)

Fresh brain tissue was taken from the ischemic cortex, cut into 1 mm^3^ size cubes and fixed in 1% freshly made paraformaldehyde with 2.5% glutaraldehyde for 24 h. Samples were fixed in 1% osmium tetroxide for 2 h and dehydrated in graded ethanol and embedded in araldite. Sections were cut at 50 nm and stained with uranyl acetate and lead citrate. Finally, the ultrastructures of the pyramidal cells, astrocyte, and the blood brain barrier (BBB) were observed with Tecnai 12 transmission electron microscope (Philips, Netherlands).

### 2.9. Quantitative Real-Time PCR

At 24 h after MCAO, rats were sacrificed and the ischemic cortex was dissected. Total RNA was extracted using TRIzol reagent (Invitrogen, Carlsbad, CA) according to the manufacturer's protocol. cDNA synthesis was performed using random hexamer primers and the TaqMan reverse transcription kit (Applied Biosystems, Foster City, CA, USA). Samples were subjected to real-time PCR analysis on a 7500 Sequence Detection System (Applied Biosystems) in accordance with the manufacturer's instructions. The primers and probes for rat glutamylcysteine synthetase high subunit (*GCSh*), glutamylcysteine synthetase light subunit (*GCSl*), nuclear factor erythroid 2-related factor 2 (*Nrf2*), tumor necrosis factor-*α* (*TNF-α*), interleukin-1*β* (*IL-1β*), interleukin-6 (*IL-6*), and* GAPDH* were designed using Primer Express 3.0 software (Applied Biosystems) based on respective GeneBank accession number. All the primers used were listed in Table 1.* GAPDH* was used as an internal control. The absolute quantities of each mRNA were calculated according to respective standard curve. Each sample was assayed in triplicate.

### 2.10. Measurement of Glutathione and Glutathione Peroxidase

The blood samples were centrifuged at 3,000 g/min at 4°C for 15 min, and serum was extracted and stored at −80°C until analyzed. The glutathione (GSH) and glutathione peroxidase (GSH-Px) activities in the serum were measured using commercial kits (Jiancheng Bioengineering Institute, Nanjing, Jiangsu, China). The activities of GSH and GSH-Px were expressed as g/L and units, respectively.

### 2.11. Enzyme-Linked Immunosorbent Assay (ELISA)

Blood samples were collected at 24 h after MCAO. Whole blood was centrifuged (13000 ×g for 15 min) and supernatants were collected to determine the level of TNF-*α*, IL-1*β*, and IL-6 in serum by available quantitative sandwich ELISA kits (R&D, USA). All use of ELISA kits was in strict accordance with the manufacturer's protocols. The concentrations of the samples were calculated according to the standard curve. The serum TNF-*α*, IL-1*β*, and IL-6 levels were all expressed as ng/L.

### 2.12. Immunohistochemistry Analysis

The immunohistochemistry staining was performed to evaluate the Bax, Bcl-2, Nrf2, and N-methyl-d-aspartate (NMDA) receptors 2A (NR2A) and 2B (NR2B) expression in brain. The hippocampal tissues were separated and fixed in 4% paraformaldehyde overnight at room temperature. The 5 *μ*m thick sections were deparaffinized and treated with 3% H_2_O_2_ methanol solution to eliminate endogenous peroxidase activity followed by blocking with 5% goat serum in tris-buffered saline. Then, the sections were incubated with anti-rat Bax (1 : 150), Bcl-2 (1 : 200), Nrf2 (1 : 100), NR2A (1 : 150), and NR2B (1 : 150) rabbit antibodies (Abcam, Cambridge, UK) in 0.01 mol/L PBS overnight at 4°C. After a PBS wash, the sections were incubated with horseradish peroxidase-conjugated goat antibodies against rabbit as the second antibody at 37°C for 30 min. After the sections were stained with diaminobenzidine kit (Zhongshan Goldenbridge Biotechnology, Beijing, China), images were acquired using a light microscope (Leica DM4000, Germany) at 400x magnification. The morphometric examination was performed in a blinded manner by two independent investigators. For each section, five visual fields were chosen at random for statistical analysis. Results were expressed as the mean number of the positive cells.

### 2.13. Statistical Analysis

All data are expressed as mean ± standard error of the mean (SEM). Statistical significance was assessed using one-way analysis of variance (ANOVA) followed by Tukey's multiple comparison tests for multiple comparisons. Values of *P* < 0.05 were considered statistically significant. All statistical analyses were performed using GraphPad Prism v5.0 (GraphPad Software, La Jolla, CA, USA).

## 3. Results 

### 3.1. Effects of EA on the Neurological Deficits and Brain Water Content after I/R

**Figure 1:**
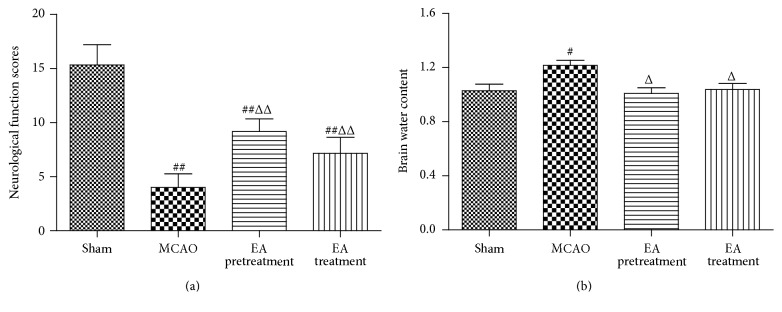
EA improves the neurological function scores and attenuates brain water content in MCAO rats. (a) Neurological function scores at 24 h after reperfusion (*n* = 6 animals per group). Rats receiving EA pretreatment and EA treatment showed significant improvement in neurological function compared with the MCAO model groups. (b) Both EA pretreatment and EA treatment significantly reduced brain water weight compared with the MCAO group (*n* = 6 animals per group). Data are represented as mean ± SEM. ^#^ *P* < 0.05 and ^##^ *P* < 0.01 versus Sham; ^Δ^ *P* < 0.05 and ^ΔΔ^ *P* < 0.01 versus MCAO. EA, electroacupuncture; MCAO, middle cerebral artery occlusion.

To investigate whether EA can influence neurological function in MCAO model rats, neurological testing was performed. After 2 h of ischemia followed by 24 h of reperfusion, rats subjected to MCAO showed significant motor behavioral deficits. Neurological function scores were significantly decreased in the MCAO group (*P* < 0.01, Figure 1(a)). Rats in both EA pretreatment and EA treatment group showed significant improvements in neurological function scores compared with MCAO group (*P* < 0.01, Figure 1(a)). Furthermore, brain water content was determined to assess brain edema in both ipsilateral and contralateral hemispheres of all the groups. Brain water content was remarkably increased in the ipsilateral hemisphere in the MCAO group (*P* < 0.05, Figure 1(b)). In contrast, EA pretreatment or EA treatment after MCAO significantly decreased brain water content in comparison with the MCAO group (*P* < 0.05, Figure 1(b)).

### 3.2. Effects of EA on Infarct Volumes in Ischemic Brains

**Figure 2:**
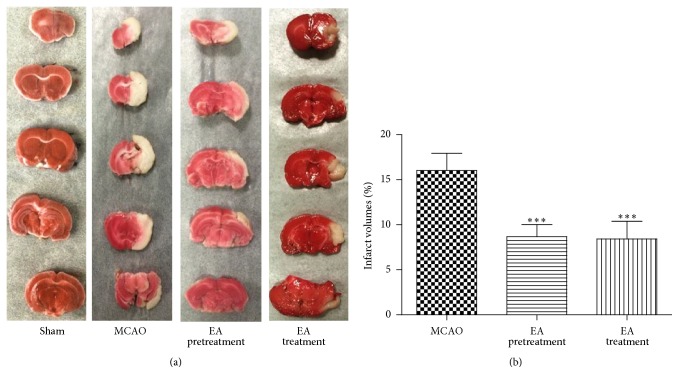
Observation of infarction volume: (a) the representative 2,3,5-triphenyltetrazolium chloride (TTC) staining (*n* = 6 animals per group), ischemic area being white and intact area stained red. (b) Percentage of infarct volume of the cerebral infarct in the rat brain (*n* = 6 animals per group). Brain tissues displayed obvious infarction in MCAO group compared to Sham-operated group. Both EA pretreatment and EA treatment groups showed a tendency of decrease in infarction volume compared to MCAO group. Results are expressed as mean ± SEM. ^*∗∗∗*^ *P* < 0.001 versus MCAO.

Infarct volume, as a measure of stroke severity, was also determined in the different groups. Extensive infarction was detected by TTC staining in the cerebral cortex in rats subjected to MCAO (Figure 2(a)). Rats pretreated with EA and treated with EA had significantly smaller infarct volumes than those in the MCAO group (*P* < 0.001, Figure 2(b)), confirming the neuroprotective effect of EA against cerebral I/R injury.

### 3.3. EA Attenuates Cerebral Damage

**Figure 3:**
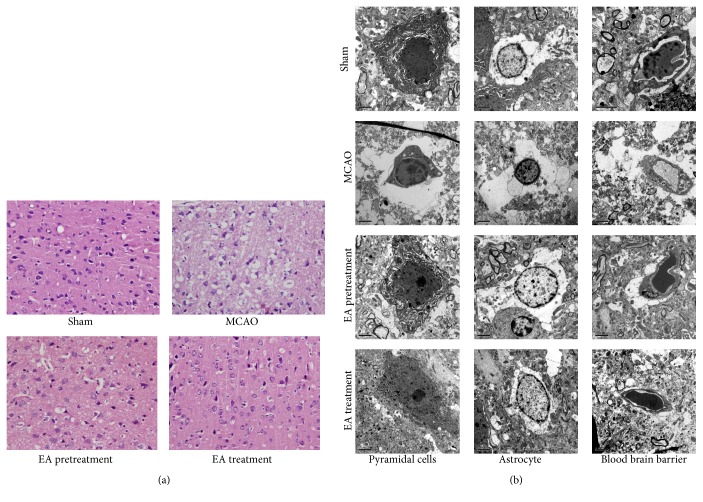
Histological examination of the ischemic cortex tissues of rats was evaluated by HE staining and TEM analysis. (a) Representative images of HE staining performed on sections from the ischemic cortex at 24 h after reperfusion in Sham, MCAO, EA pretreatment, and EA treatment groups (*n* = 6 animals per group). Ischemic cortex sections obtained from injured cerebral hemispheres were stained with haematoxylin and eosin and observed using Olympus microscope (×400). (b) Ultrastructure changes by transmission electron microscope (×5800). Graphs showing the ultrastructural changes of pyramidal cells, astrocyte, and blood brain barrier (BBB) in different groups (*n* = 6 animals per group).

HE staining was performed to observe the morphological changes, as shown in Figure 3(a). In the Sham group, there was no obvious pathological change in cortex. The arrangement of pyramidal cells was close and orderly, neurons kept arranged well, the nuclei were centered with clear staining, and the cytoplasm was abundant. In contrast, the ischemic cortex in the MCAO was damaged seriously. Neurons were significantly degenerated and necrotic, and their arrangement was disordered and sparse. There was nerve cells loss, and edema and deformation were visible with nuclear pyknosis, deep staining, and unclear nucleolus. However, EA pretreatment and EA treatment can obviously decrease the extent of damage induced by MCAO, and an edema and loss and deformation of the nerve cells were alleviated in cortex of rats. In addition, the number of normal neurons was markedly increased as well.

The neuroprotective effect of EA against cerebral ischemic damage was also supported by TEM. As shown in Figure 3(b), in the Sham group, the pyramidal cells were featured by elliptical cell nucleus, even chromatin, clear nucleolus, and cytoplasm. Moreover, there were many Golgi bodies, rough endoplasmic reticulum, and mitochondria with intact cristae in pyramidal cells. The vascular endothelial cells had smooth and flat surfaces, and the endothelia, basement membranes, and foot processes were in close contact. In contrast to Sham group, the pyramidal cells and astrocytes in MCAO showed shrunken nucleus, swollen cell organelles, chromatin condensation, and marginalization and formation of apoptotic bodies. Edema vacuoles around the minute vessels were observed outside the cells. The endothelial cells were swollen, and the thickened basement membrane was not well organized. In the treatment group, the damage to the neurons was alleviated compared to the model group. In EA pretreatment and treatment group, the presence of edema in organelle, cytoplasm, and vascular anomaly was obviously reduced, and the shrunken nucleus in nerve cells and astrocytes were alleviated. The vascular endothelial cells and the basement membrane exhibited smooth and intact surfaces with clear layers.

### 3.4. EA Inhibits Apoptosis following Cerebral I/R

**Figure 4:**
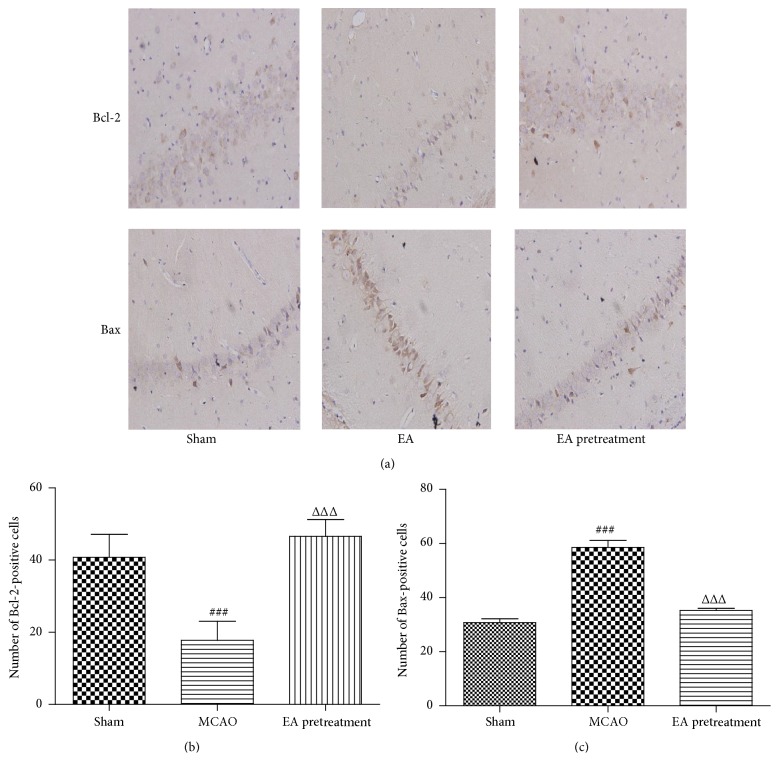
Effects of EA on the expression of Bax and Bcl-2 were investigated by immunohistochemistry assay after MCAO. (a–c) Immunohistochemistry staining for Bax or Bcl-2-positive cells in hippocampal CA1 region in different groups (*n* = 6 animals per group) (×400). Data represent mean ± SEM. ^###^ *P* < 0.001 versus Sham and ^ΔΔΔ^ *P* < 0.001 versus MCAO.

The protooncoproteins (Bcl-2 and Bax) are key regulators of the mitochondrial apoptotic pathway initiated by a variety of extracellular and intracellular stressors. To investigate whether EA could attenuate apoptosis in the hippocampal tissues after ischemia-reperfusion, we analyzed the protein expression status of Bax and Bcl-2 by immunohistochemistry staining. As shown in Figures 4(a) and 4(b), the number of Bcl-2-positive cells decreased in the ischemic hippocampal CA1 region of the MCAO rats compared with the Sham group. However, the number of Bcl-2-positive cells was significantly higher in EA pretreatment rats relative to MCAO (*P* < 0.001). Conversely, the number of Bax-positive cells markedly decreased in the EA pretreatment group compared to MCAO group (*P* < 0.001, Figures 4(a) and 4(c)). These data indicate that EA pretreatment could balance the expression of apoptosis related proteins Bcl-2 and Bax and prevent the neuronal apoptosis in hippocampus.

### 3.5. EA Promotes the Expression of Nrf2 and GCS

**Figure 5:**
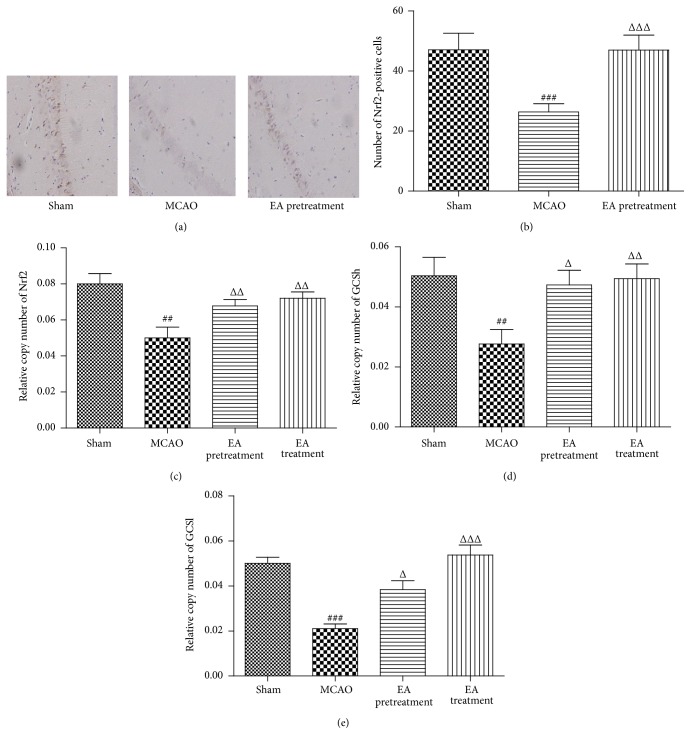
Effects of EA on the expression of Nrf2 after MCAO. (a and b) Immunohistochemistry staining for Nrf2-positive cells in hippocampal CA1 region in different groups (*n* = 6 animals per group) (×400). (c–e) Analysis of* Nrf2*,* GCSh*, and* GCSl* mRNA expression levels in cortex by real-time PCR. GAPDH was used as an internal control (*n* = 8 animals per group). Data represent mean ± SEM. ^##^ *P* < 0.01, and ^###^ *P* < 0.001 versus Sham and ^Δ^ *P* < 0.05, ^ΔΔ^ *P* < 0.01, and ^ΔΔΔ^ *P* < 0.001 versus MCAO.

Nrf2 is a key transcription factor that regulates antioxidant genes as an adaptive response to oxidative stress. To identify whether Nrf2/GCS signaling is involved in the neuroprotective effect of EA, we analyzed the expression of Nrf2 in ischemic hippocampal tissues by immunohistochemistry staining. Results showed that EA pretreatment induced a remarkable upregulation of Nrf2-positive cells in hippocampal CA1 region when compared with the MCAO group counterparts (*P* < 0.001, Figures 5(a) and 5(b)). Real-time PCR analysis at 24 h following reperfusion showed, in the ischemic hippocampal CA1 region in EA pretreatment and treatment rats, a significantly higher expression of* Nrf2* mRNA level in comparison with that of MCAO animals (*P* < 0.01, Figure 5(c)).

GCS, a heterodimer consisting of heavy (GCSh) and light (GCSl) subunits, is regulated by Nrf2 [24]. It catalyzes the rate-limiting* de novo* biosynthesis of GSH, an abundant physiological antioxidant that plays important roles in regulating oxidative stress. Here, we found that there was remarkable increase in* GCSh* and* GCSl* mRNA levels in rats treated with EA pretreatment and treatment, compared with MCAO group (*P* < 0.05, Figures 5(d) and 5(e)).

### 3.6. EA Upregulates Endogenous Antioxidant Systems following I/R

**Figure 6:**
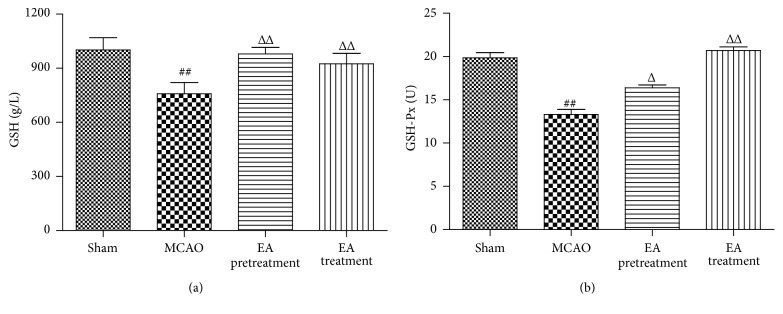
Effects of EA on the activities of GSH and GSH-Px. Treatment with EA significantly increased GSH (a) and GSH-Px (b) activities in serum compared with the MCAO group (*n* = 8 animals per group). Data represent mean ± SEM. ^##^ *P* < 0.01 versus Sham and ^Δ^ *P* < 0.05 and ^ΔΔ^ *P* < 0.01 versus MCAO.

To further understand the effect of EA on Nrf2 downstream antioxidant enzymes, we detected the activities of GSH and GSH-Px in rats' serum to examine the oxidative response at 24 h after ischemia. The activities of GSH and GSH-Px were significantly lower in the MCAO group compared with the Sham group, which was restored by EA pretreatment and treatment (*P* < 0.01, Figure 6).

### 3.7. Effects of EA on the Levels of TNF-*α*, IL-1*β*, and IL-6 in Ischemic Cortex and Serum after MCAO

**Figure 7:**
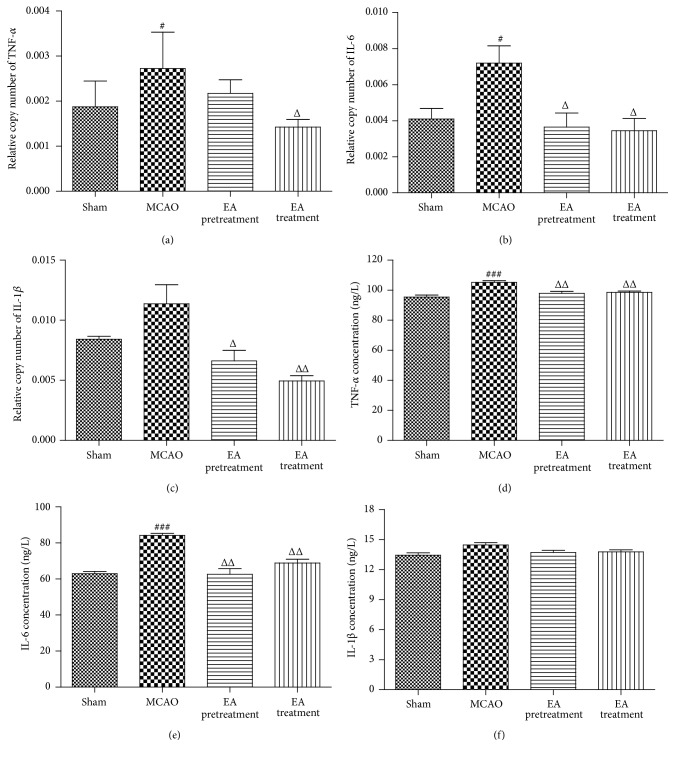
Effects of EA on the levels of TNF-*α*, IL-6, and IL-1*β* in ischemic cortex and serum after MCAO. (a–c) The levels of* TNF-α*,* IL-6*, and* IL-1β* mRNA in cortex were determined by real-time PCR (*n* = 8 animals per group). GAPDH was used as an internal control. (d–f) Protein concentrations of TNF-*α*, IL-6, and IL-1*β* in the serum in different groups (*n* = 8 animals per group). Data represent mean ± SEM. ^###^ *P* < 0.001 versus Sham and ^Δ^ *P* < 0.05 and ^ΔΔ^ *P* < 0.01 versus MCAO. ^#^ *P* < 0.05 versus Sham.

To explore whether EA could induce an anti-inflammatory pattern, we examined proinflammatory mediators in the ischemic cortex and serum after 24 h of reperfusion. Real-time PCR analysis showed a significant increase in* TNF-α* and* IL-6* in ischemic cortex 24 h after MCAO (*P* < 0.05, Figures 7(a) and 7(b)). EA treatment markedly suppressed ischemia-induced upregulation of* TNF-α*,* IL-1β*, and* IL-6* in ischemic cortex (*P* < 0.05, *P* < 0.05, and *P* < 0.01, Figures 7(a), 7(b), and 7(c)). In addition, EA pretreatment also significantly reduced the expression of* IL-1β* and* IL-6* in ischemic cortex (*P* < 0.05, Figures 7(a), 7(b), and 7(c)). Consistent with the inhibitory effect of EA pretreatment on mRNA expression, the protein concentrations of TNF-*α* and IL-6 in serum 24 h after MCAO were measured by ELISA, and the results showed a similar trend to those observed in the real-time PCR analysis (Figures 7(d) and 7(e)). EA treatment also significantly inhibited the protein concentrations of TNF-*α* and IL-6 induced by MCAO (Figures 7(d) and 7(e)).

### 3.8. Effects of EA on the Expression of NR2A and NR2B in Hippocampus

**Figure 8:**
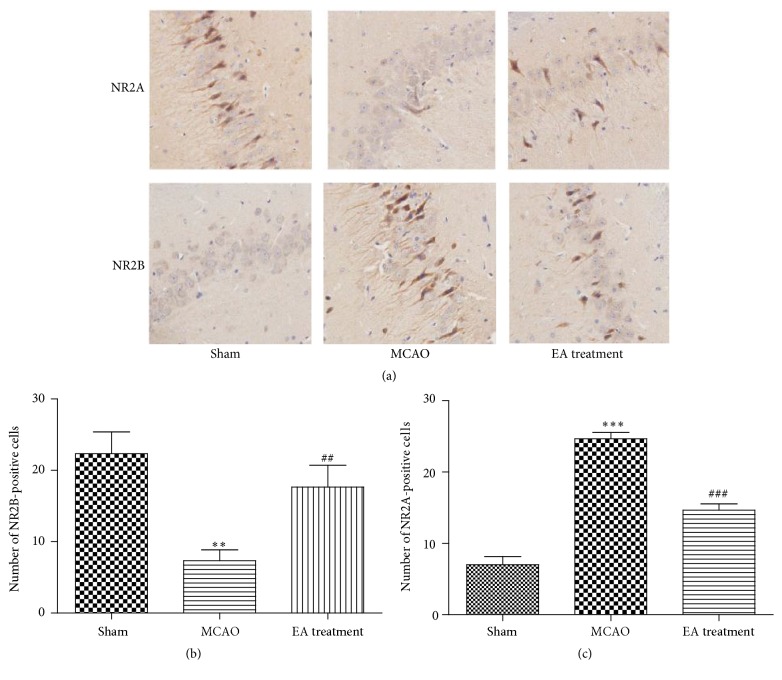
Effects of EA on expression of NR2A and NR2B after MCAO. (a–c) Immunohistochemistry staining for NR2A- and NR2B-positive cells in hippocampal CA1 region in different groups (×400) (*n* = 6 animals per group). Data represent mean ± SEM. ^*∗∗*^ *P* < 0.01 and ^*∗∗∗*^ *P* < 0.001 versus Sham and ^##^ *P* < 0.01 and ^###^ *P* < 0.001 versus MCAO.

One of the major hallmarks of cerebral ischemia is excitotoxicity. The N-methyl-d-aspartate (NMDA) receptors (NMDARs) are considered to be largely responsible for excitotoxic injury due to their high Ca^2+^ permeability. In the hippocampus and cortex, NMDARs are most prominently composed of combinations of two N1 subunits and two N2A (NR2A) and/or N2B (NR2B) subunits. However, the differential role of NR2A and NR2B subunits in excitotoxic injury was still controversial [25]. To identify the effects of EA on NR2A and NR2B, we analyzed the expression of NR2A and NR2B in hippocampal CA1 region by immunohistochemistry staining. Compared with Sham group, the numbers of NR2A-positive cells were significantly lower and the numbers of NR2B-positive cells were significantly higher in MCAO group (Figures 8(a), 8(b), and 8(c)). However, EA treatment induced a remarkable upregulation of NR2A-positive cells and downregulation of NR2B-positive cells in hippocampal CA1 region when compared with the MCAO group counterparts (Figures 8(a), 8(b), and 8(c)). In this regard, EA may alleviate the excitotoxicity which was medicated by NMDAR and attenuate I/R injury via affecting the whole activity of NMDARs complex (NR2A/NR2B).

## 4. Discussion

The present study analyzed the preventive and therapeutic potential of EA and its underlying mechanisms. Notably, we found that neuroprotective effect of EA was associated with the inhibition of apoptosis, preservation of antioxidative enzymes, reduction of inflammatory mediators, and alleviation of excitotoxicity. Taken together, these results indicated that EA could be a promising tool for complementary therapy in stroke.

Acupuncture represents an integral part of the traditional Chinese medical system. It could regulate body homeostasis and induce enormous physiological potential with minority energy stimulation. As evidences given by recent extensive reports, the beneficial effects of EA on brain ischemic damage in vivo or in vitro mainly focus on antiapoptotic [26], anti-inflammatory [27], and neuron protection [28]. Our study indicates that the underlying mechanism of EA against cerebral I/R injury is a multiple interaction which may involve the inhibition of apoptosis, preservation of antioxidative system, reduction of inflammatory response, and attenuation of excitotoxicity in MCAO rats.

Apoptosis is one of the major causes of cerebral I/R injury. Neuronal death or survival is dependent on the balance between proapoptotic (Bax) and antiapoptotic (Bcl-2) proteins during cerebral ischemia [29, 30]. It is well known that the increase in brain damage is associated with increased apoptosis as indicated by increased levels of Bax and decreased levels of Bcl-2. A variety of evidences showed that induction of Bcl-2 expression was believed to be protective against ischemic insult. For example, antisense knockdown of endogenous Bcl-2 mRNA exacerbated cerebral ischemic injury in rats and blocked the neuroprotection afforded by ischemic preconditioning [31, 32]. In the present study, we found that the brains of MCAO rats showed obvious apoptotic morphology with dramatically decreased levels of Bcl-2 and increased levels of Bax. This is consistent with previous reports regarding the production of apoptosis [33, 34]. EA could decrease the number of apoptotic cells, upregulate Bcl-2, and downregulate Bax in the ischemic brain. Further studies are required to dissect the detailed mechanisms underlying the regulation of apoptosis.

Extensive studies have been carried out to investigate the immune mechanism of cerebral ischemia. Experiments indicated that inflammatory reactions played crucial roles in brain damage after cerebral I/R injury [35]. During cerebral ischemia, proinflammatory mediators such as TNF-*α*, IL-1*β*, and IL-6 are excessively produced by a variety of activated cell types, including microglia, endothelial cells, astrocytes, and neuron cells, which finally exacerbated neuronal injury [36]. Therefore, it is reasonable to speculate that pharmacological alleviation of inflammatory response may be a beneficial choice for stroke therapy [37]. EA has been reported to exert anti-inflammatory effects in several infectious and noninfectious disease models, such as passive endotoxemia, cutaneous anaphylaxis, spinal cord injury, and amyotrophic lateral sclerosis [38–41]. Lan et al. observed that EA decreased the levels of TNF-*α*, IL-1*β*, and IL-6 through the suppression of the toll-like receptor 4 and nuclear factor-kappa B (TLR4/NF-*κ*B) signaling pathway in cerebral I/R injured rats [42]. In our study, we also found that EA decreased the expression of TNF-*α*, IL-1*β*, and IL-6 in serum and brain tissues. Our study revealed that EA could modulate the systemic and local inflammatory reaction in MCAO rats.

The role of oxidative stress in pathogenesis of neuronal death after cerebral ischemia has emerged as an attractive field [43]. Nrf2 is a key regulator of antioxidant and antioxidative genes including *γ*-GCS, heme oxygenase 1 (HO-1), and superoxide dismutase (SOD) which plays a vital role in antagonizing oxidative stress [44]. More importantly, Nrf2 is becoming a promising therapeutic target for neuroprotection [45]. In addition, EA could exert antioxidant properties to counteract the oxidative stress in Parkinson's disease through activation of Nrf2 pathway [13]. Recent studies also reported that intervention of Nrf2 defense pathway could facilitate ischemic brain injury alleviation [46, 47]. In this study, EA significantly increased the expression of Nrf2 in hippocampus at 24 h after MCAO. Our study also demonstrated that EA treatment could upregulate *γ*-GCS, the rate-limiting enzyme in GSH biosynthesis. Given that these genes were regulated by Nrf2, we deduced that neuroprotective effect of EA might be associated with the upregulation of Nrf2 and GCS.

GSH-Px, GSH, and SOD, indicators of the rate and extent of oxidative stress, could provide first line of protection from ischemic cerebral injury [48]. EA induced neuroprotection against cerebral ischemia through reducing oxidative stress, which may provide new mechanisms [49, 50]. To explore the possible mechanism of EA on the relief of oxidative stress induced by I/R injury, we also investigated the EA-dependent effects on GSH-Px and GSH in MCAO rats. In the present study, increased activity of antioxidant enzymes can be found in MCAO rats. In this regard, our results have provided evidence for EA's antioxidative activity after cerebral I/R.

Since EA has several advantages, such as economy, convenience, and few side effects, it has been widely applied in stroke patients and in animal stroke models [16, 51]. Preconditioning or pretreatment, potent endogenous protective response, activates several endogenous signaling pathways that result in tolerance against ischemia [52]. Recently, numerous studies have shown that EA pretreatment reduced focal cerebral ischemia in a manner mimicking the ischemia pretreatment [51, 53, 54]. Our study demonstrated that EA pretreatment at* Baihui* (GV20) and* Dazhui* (GV14) for 30 min a day for five days could reduce infarct volumes and improve neurological function after 24 h occlusion. These findings indicate that EA pretreatment could also induce tolerance to cerebral ischemic insult. Meanwhile, the design of EA pretreatment in our study embodies the academic theory of “preventive treatment of disease,” which provides enormous guidance for disease preventions. Although it has been known that EA pretreatment can induce tolerance to ischemic brain injury [17, 55], the molecular mechanisms that contribute to brain ischemia tolerance by EA pretreatment remain poorly understood. In this study, we found that EA pretreatment attenuated cerebral ischemia and reperfusion injury in MCAO rat via modulation of apoptosis, inflammation, and oxidative stress.

Excitotoxicity, a kind of neurotoxicity mediated by glutamate, provides link between ischemia and neuronal death, and intervention of the relevant molecules that result in excitotoxicity can prevent stroke damage [56]. Glutamate exerts its function by activating the NMDAR which has been shown to be a critical factor in neuronal damage following ischemia-reperfusion insults [57]. NR2A is predominantly located at synapses, whereas NR2B is mainly found at extrasynaptic locations. However, there was an opposing action of the NR2A and NR2B subunits in mediating cell death and cell survival [58–60]. These evidences indicate that the NR2A subunit produces prosurvival activity, whereas the NR2B subunit leads to a prodeath signal. There are few reports about the effects of EA on expression of NMDAR in stroke. Reports showed that EA reversed the high NR1 subunit expression in a MCAO rat [61, 62]. Our data showed that EA treatment induced a remarkable upregulation of NR2A and downregulation of NR2B in hippocampus. Hence, EA may alleviate the excitotoxicity which was medicated by NMDAR and attenuate I/R injury via affecting the activity of NR2A/NR2B. Sun et al. reported that NR2B was more lethal than those containing NR2A. Moreover, prodeath signaling pathways mediated by neuronal nitric oxide synthase (nNOS), phosphatase and tensin homolog located on chromosome 10 (PTEN), and calcium/calmodulin-dependent protein kinase II (CaMKII) have been linked to NR2B activation [59]. Therefore, the effects of the signaling pathways that activated NR2B are worthy of consideration.

Several studies have shown that EA stimulation potentially provided neuroprotection effects against cerebral I/R injury at different acupoints and at various frequencies. For instance, experimental studies in rats have shown that EA stimulation at the* Baihui* acupoint (2/15 Hz) displayed antiapoptotic effects by increasing the Bcl-2 expression [63, 64]. Tian et al. demonstrated that EA stimulation at the* Baihui*,* Mingmen*, and* Zusanli* acupoints (30/50 Hz) provided neuroprotection in MCAO rats [65]. Moreover, Kim et al. have reported that EA at* Baihui* and* Dazhui* acupoints elicited neuroprotection against cerebral I/R injury [66]. In this study, we chose the* Baihui* and* Dazhui* acupoints according to our practice experience.* Baihui* could affect nerve and periosteum efficiency, dredge and activate* Du* meridian, and revive brain.* Dazhui* could activate blood circulation to dissipate blood stasis and also activate brain function to cause resuscitation. Our findings thus suggest that EA stimulation at* Baihui* and* Dazhui* acupoints, at a frequency of 2/15 Hz, exerts neuroprotective effects against cerebral I/R injury.

Recently, Tao et al. reported that EA alleviated neurological deficits possibly by promoting the proliferation and differentiation of nerve stem cells (NSC) [67]. Extra experiments are required to confirm the influence of EA on the proliferation and differentiation of NSC and neurogenesis. Moreover, additional targets of EA may be relevant for stroke. Future studies need to knock down these targets to evaluate their relevance for protection.

In conclusion, the present results indicate that EA pretreatment or treatment may induce a neuroprotection in transient MCAO rats. The underlying mechanisms were associated with the inhibition of apoptosis, preservation of antioxidative systems, reduction of inflammatory mediators, and alleviation of excitotoxicity.

## Figures and Tables

**Table 1:** All the primers information used in quantitative real-time PCR.

Gene		Primer sequence
*GCSh*	Forward primer	5′-TATCTGCCCAATTGTTATGGCTTT-3′
	Reverse primer	5′-TCCTCCCGTGTTCTATCATCTACA-3′
	Probe	5′-CATCGCCATTTTACCGAGGCTACGTG-3′

*GCSl*	Forward primer	5′-GGGCACAGGTAAAACCCAATAG-3′
	Reverse primer	5′-TTGGGTCATTGTGAGTCAGTAGCT-3′
	Probe	5′-TTAATCTTGCCTCCTGCTGTGTGATGCC-3′

*Nrf2*	Forward primer	5′-CCATTCCCGAGTTACAGTGTCTT-3′
	Reverse primer	5′-GATCGATGAGTAAAAATGGTAATTGC-3′
	Probe	5′-CAGCCCAGAGGCCACACTGACAGA-3′

*IL-1β*	Forward primer	5′-TGTGATGAAAGACGGCACAC-3′
	Reverse primer	5′-CTTCTTCTTTGGGTATTGTTTGG-3′
	Probe	5′-AGCTGGAG-3′

*TNF-α*	Forward primer	5′-TGAACTTCGGGGTGATCG-3′
	Reverse primer	5′-GGGCTTGTCACTCGAGTTTT-3′
	Probe	5′-AGGAGGAG-3′

*IL-6*	Forward primer	5′-CCCTTCAGGAACAGCTATGAA-3′
	Reverse primer	5′-ACAACATCAGTCCCAAGAAGG-3′
	Probe	5′-CCAGCCAG-3′

*GAPDH*	Forward primer	5′-CCTCAAGATTGTCAGCAATGCA-3′
	Reverse primer	5′-TGGCAGTGATGGCATGGA-3′
	Probe	5′-CACCACCAACTGCTTAGCCCCCCT-3′
